# A rapid and sensitive assay of intercellular coupling by voltage imaging of gap junction networks

**DOI:** 10.1186/1478-811X-11-78

**Published:** 2013-10-21

**Authors:** Federico Ceriani, Fabio Mammano

**Affiliations:** 1 Dipartimento di Fisica e Astronomia “G. Galilei”, Università di Padova, Padova 35131, Italy; 2 Istituto Veneto di Medicina Molecolare, Fondazione per la Ricerca Biomedica Avanzata, Via G. Orus, 2, Padova 35129, Italy; 3 Istituto di Neuroscienze, Consiglio Nazionale delle Ricerche, Padova 35131, Italy

**Keywords:** Connexins, Electrical coupling, Dye coupling, Genetic deafness, Voltage sensitive dye, Digital phase−sensitive detector

## Abstract

**Background:**

A variety of mechanisms that govern connexin channel gating and permeability regulate coupling in gap junction networks. Mutations in connexin genes have been linked to several pathologies, including cardiovascular anomalies, peripheral neuropathy, skin disorders, cataracts and deafness. Gap junction coupling and its patho–physiological alterations are commonly assayed by microinjection experiments with fluorescent tracers, which typically require several minutes to allow dye transfer to a limited number of cells. Comparable or longer time intervals are required by fluorescence recovery after photobleaching experiments. Paired electrophysiological recordings have excellent time resolution but provide extremely limited spatial information regarding network connectivity.

**Results:**

Here, we developed a rapid and sensitive method to assay gap junction communication using a combination of single cell electrophysiology, large–scale optical recordings and a digital phase–sensitive detector to extract signals with a known frequency from Vf2.1.Cl, a novel fluorescent sensor of plasma membrane potential. Tests performed in HeLa cell cultures confirmed that suitably encoded Vf2.1.Cl signals remained confined within the network of cells visibly interconnected by fluorescently tagged gap junction channels. We used this method to visualize instantly intercellular connectivity over the whole field of view (hundreds of cells) in cochlear organotypic cultures from postnatal mice. A simple resistive network model reproduced accurately the spatial dependence of the electrical signals throughout the cellular network. Our data suggest that each pair of cochlear non−sensory cells of the lesser epithelial ridge is coupled by ~1500 gap junction channels, on average. Junctional conductance was reduced by 14% in cochlear cultures harboring the T5M mutation of connexin30, which induces a moderate hearing loss in connexin30^T5M/T5M^ knock–in mice, and by 91% in cultures from connexin30^−/−^ mice, which are profoundly deaf.

**Conclusions:**

Our methodology allows greater sensitivity (defined as the minimum magnitude of input signal required to produce a specified output signal having a specified signal−to−noise ratio) and better time resolution compared to classical tracer–based techniques. It permitted us to dynamically visualize intercellular connectivity down to the 10th order in non−sensory cell networks of the developing cochlea. We believe that our approach is of general interest and can be seamlessly extended to a variety of biological systems, as well as to other connexin−related disease conditions.

## Background

Cell–cell communication mediated by gap junctions is crucial to a variety of cellular functions, including the regulation of cell growth, differentiation and development [1]. In electrically excitable cells, gap junctions provide low–resistance pathways, traditionally referred to as electrical synapses, and permit transmission of electrical signals between adjacent cells. In the brain, electrical synapses have been shown to be important for enabling and detecting neuronal synchrony [2,3] and to regulate lineage–dependent microcircuit assembly [4]. In the heart, the ability to synchronize groups of cells is crucial to achieve a coordinated mechanical output [5,6]. In non–excitable cells, gap junctions permit to share metabolic demands across groups of cells, enable the exchange of signaling molecules [7,8] and the spatial buffering of potassium ions [9].

Virtually all cells in solid tissues are coupled by gap junctions [1], thus it is not surprising that mutations in connexin genes have been linked to a variety of human diseases, including cardiovascular anomalies, peripheral neuropathy, skin disorders, cataracts and deafness [10-12]. Gap junction channels in the mammalian cochlea, the site of the sense of hearing, are formed primarily by connexin26 and connexin30 proteins encoded by nonsyndromic hearing loss and deafness (DNFB1) genes *GJB2* and *GJB6,* respectively [13]. Cochlear connexins are expressed very early on in development and interconnect virtually all types of non−sensory cells [14-16]. Morphological analysis of cochleae from different strains of mice with (targeted) ablation of connexin26 or connexin30 provide evidence of incomplete or arrested development ensuing in defects of hearing acquisition [12].

The most widely used approach to monitor intercellular communication employs optical methods to track the movement of tracer molecules between neighboring cells. However, the sensitivity of this technique depends on the junctional permeability of the tracer employed, which varies significantly with the size of the permeant molecule and the type of gap junction channels. Sensitivity can be increased by prolonging the loading time or by employing smaller tracer molecules (e.g. serotonin [17]).

Here, we used cochlear organotypic cultures to unravel the potential of Vf2.1.Cl, a member of the novel VoltageFluor (VF) family of fluorescent sensors [18]. VF dyes detect voltage changes by modulation of photo–induced electron transfer (PeT) from an electron donor through a synthetic molecular wire to a fluorophore. They have large, linear, turn–on fluorescence responses to depolarizing steps (20–27% fluorescence change per 100 mV), fast kinetics (τ << 140 μs) and negligible capacitative loading. We exploited the Vf2.1.Cl voltage sensitive dye [18] to probe dynamically the extent of gap junction coupling by a combination of single cell electrophysiology, large scale optical recordings and a digital phase–sensitive detector of fluorescence signals. Our method is readily applicable to a variety of cellular systems, as it requires only a patch–clamp amplifier to inject sinusoidal electrical signals at fixed frequency and amplitude in a single cell and a fluorescence microscope to track optically the VF dye response at the frequency of the stimulus throughout the network.

## Results

### *In–situ* calibration of the Vf2.1.Cl voltage sensitive dye

**Figure 1:**
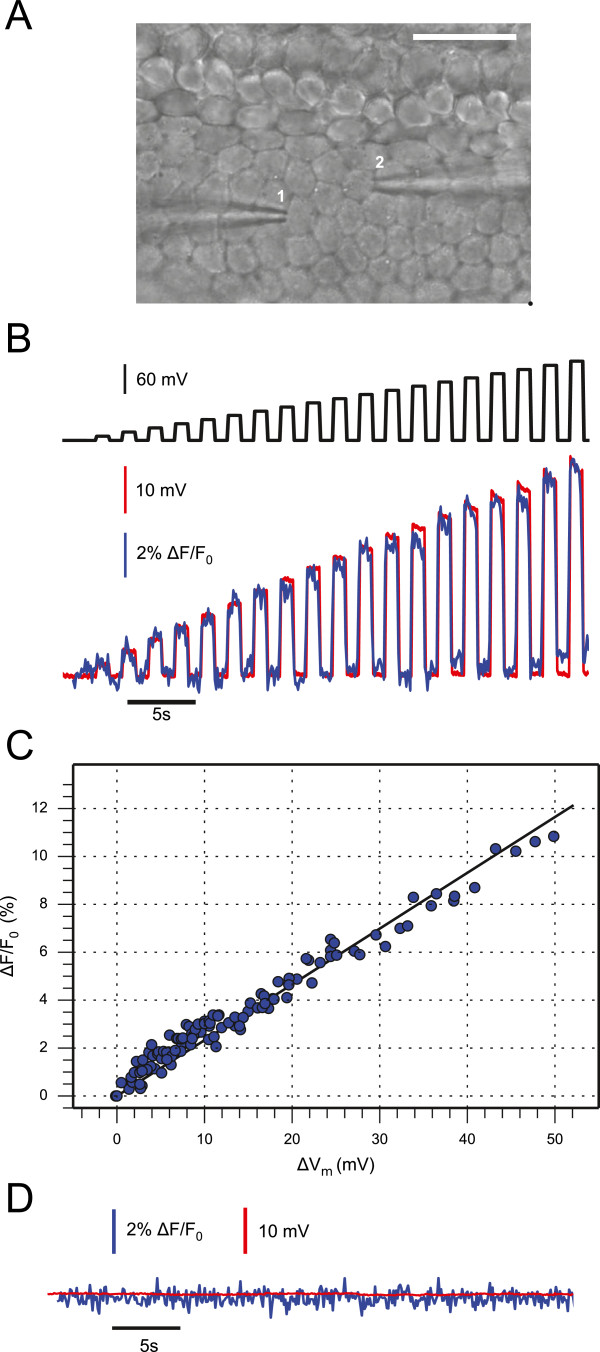
**Calibration of Vf2.1.Cl voltage responses by paired patch clamp recordings in cochlear organotypic cultures. (A)** Differential interference contrast (DIC) image showing two patch clamp pipettes, each one sealed to a non−sensory cell of the lesser epithelial ridge; scale bar, 25 μm. **(B)** Representative traces showing simultaneous membrane potential (red) and Vf2.1.Cl fluorescence (blue) from cell 2, in the neighborhood of the stimulated cell (cell 1); the black trace (top) represents the waveform of the stimulus delivered by the patch clamp amplifier connected to cell 1. **(C)** Fractional fluorescence signal change (Δ*F/F*_0_) vs. membrane potential change Δ*V*_*m*_ (both signals are from cell 2); dots are individual measurements from *n* = 5 cells in 3 cultures; the straight line is a linear fit to the data. **(D)** Both voltage and fluorescence responses of cell 2 were suppressed after incubating the culture for 20 minutes in 100 μM carbenoxolone (CBX).

Organotypic cultures of cochlear explants from postnatal mice permit to investigate the patho–physiology of gap–junction–mediated intercellular signaling in a readily accessible whole–organ context [19-24]. In order to calibrate the voltage response of the fluorescent sensor in our experimental conditions (see Methods), we loaded organotypic cultures from wild type mice, euthanized at postnatal day 5 (P5), with Vf2.1.Cl. We then performed paired whole–cell patch clamp recordings from cochlear non−sensory cells of the lesser epithelial ridge. We stepped the voltage *V*_0_ of the patch clamp amplifier connected to one cell (cell 1, Figure 1A) in 10 mV increments (Figure 1B, black trace) from the zero current potential (−61±2 mV, *n* = 15 cells) while monitoring the membrane potential (*V*_*m*_) (Figure 1B, red trace) of a nearby cell (cell 2, Figure 1A) maintained under current–clamp conditions with a second amplifier. At the same time, we measured Vf2.1.Cl fluorescence emission (*F*) from cell 2 (Figure 1B, blue trace). Data in Figure 1B,C highlight a linear relationship between the change in membrane potential (Δ*V*_*m*_) and the corresponding fractional change (Δ*F/F*_0_) in Vf2.1.Cl fluorescence emission. Note that both Δ*F/F*_0_ and Δ*V*_*m*_ were detected from cell 2. The correlation coefficient between Δ*F/F*_0_ and Δ*V*_*m*_ was *R* = 0.98 (*n* = 5 paired recordings in 3 cultures) and a linear fit to the data (Figure 1C, solid line) yielded a responsivity (slope) *m* = 0.23 ± 0.03 Δ*F/F*_0_*/*mV (i.e. 23 ± 3% per 100 mV). Both Δ*V*_*m*_ and Δ*F/F*_0_ responses were suppressed after incubating the culture for 20 minutes in 100 μM carbenoxolone (CBX, Figure 1D), a non−selective blocker of gap junction channels [25].

Based on this calibration, we estimated optically the voltage step in cell 1 (Δ*V*_1_) corresponding to a given voltage command Δ*V*_0_ delivered by the patch clamp amplifier. On average, Δ*V*_0_ = 70 mV yielded a Δ*V*_1_ = 22 ± 4 mV (*n* = 5) in wild type cultures. We then derived the access resistance of the patch pipette connected to cell 1 as *R*_*a*_ = Δ(*V*_0_−*V*_1_)/Δ*I*, where Δ*I* = 6.8 ± 1.1 nA (*n* = 5) is the current step measured by the amplifier. The value we obtained, *R*_*a*_ = 7.8 ± 0.9 MΩ (*n* = 5), is in excellent agreement with the estimate provided by the membrane test of the patch clamp software, *R*_*a*(patch)_ = 7.5 ± 1.2 MΩ (*n* = 5).

### A digital phase–sensitive detector of Vf2.1.Cl signals visualizes and quantifies network connectivity

Paired electrophysiological recordings, such as those in Figure 1, have excellent time resolution but provide extremely limited spatial information regarding network connectivity. The main goal of the present study was to visualize rapidly network connectivity using large−scale optical recordings of Vf2.1.Cl florescence in different preparations and experimental conditions. The calibration procedure reported in Figure 1 yielded maximal fluorescence changes Δ*F/F*_0_ in cell 2, close to cell 1, which rarely exceeded 10%. Electrical signals spreading passively through a resistive network are expected to attenuate rapidly with distance from the source (i.e. cell 1) and fluctuations due to photon shot noise hamper their detection [26]. We sought to overcome these limitations by the following procedure.

**Figure 2:**
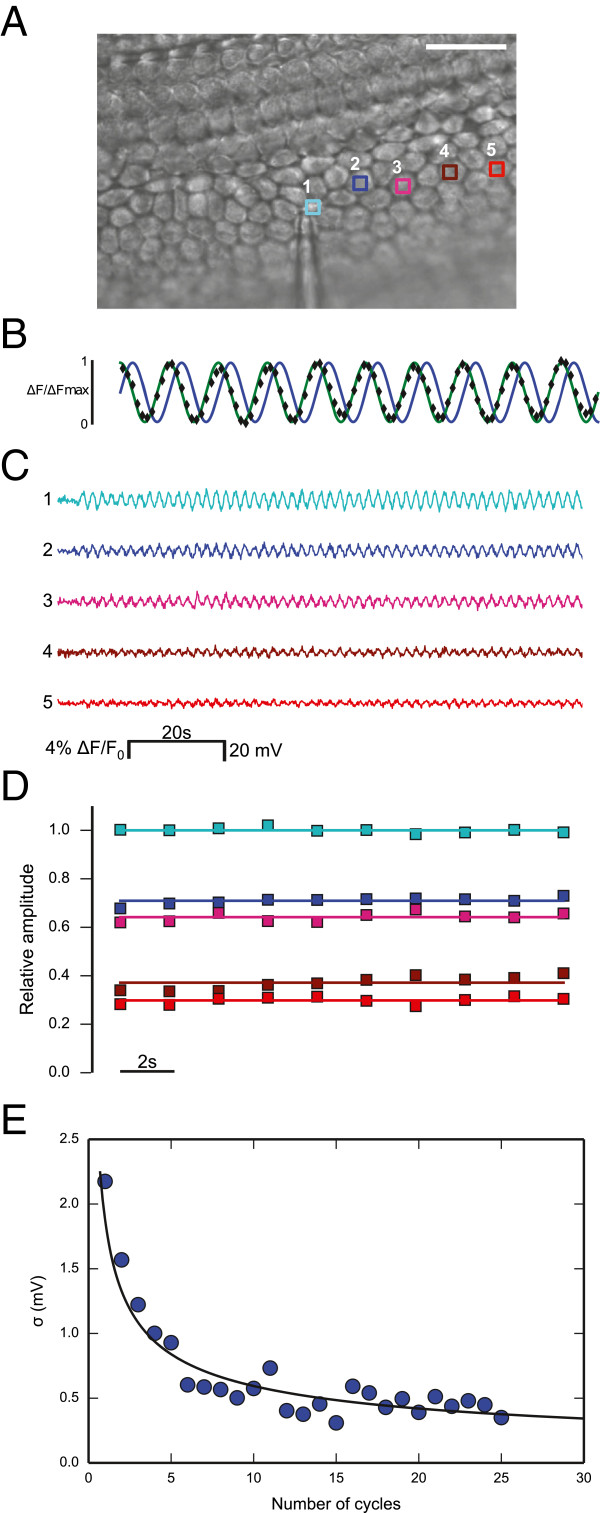
**Illustrating phase–sensitive detection of Vf2.1.Cl fluorescence responses. (A)** DIC image showing a single patch clamp pipette sealed to a non−sensory cell of the lesser epithelial ridge (cell 1, zero current potential −66 mV); scale bar, 25 μm. **(B)** Black diamonds: normalized optical signals from a specific cell network location; green trace: unit amplitude carrier wave delivered to cell 1; blue trace: its phase–shifted counterpart used in the computation of signal amplitude (see Methods). **(C)** Calibrated optical responses from the five regions of interest (ROIs) shown in **(A)** during a typical stimulation protocol. A low order polynomial fit was subtracted to the raw traces to compensate for the effects of photobleaching (see Methods). **(D)** Relative amplitude signals derived by integrating traces shown in **(C)** over a single carrier wave cycle (*N* = 1). **(E)** The standard deviation *σ* of the single pixel amplitude signal *A*(*x,y*) is plotted against the number *N* of integration cycles (see Methods); the black solid line is a least square fit to the data with the function *σ*_1_/*N*^½^ where *σ*_1_ = 1.9 mV.

We loaded cochlear organotypic cultures from P5 mice with the Vf2.1.Cl dye and delivered a sinusoidal voltage command, also named carrier wave (frequency ν = 0.5 Hz, amplitude 35 mV) to the patch clamp amplifier connected to one cell of the network (cell 1, Figure 2A). In wild type cultures, this stimulation elicited instantly sinusoidal optical signals of Vf2.1.Cl fluorescence at the frequency ν of the carrier wave (reference frequency) in virtually all cells of the network within the field of view (Additional file 1: Movie S1). We then used the off−line digital phase–sensitive detector (also known as lock–in amplifier) described in the Methods to extract Vf2.1.Cl signal amplitude *A*(*x,y*) at each network location (*x,y*) at the reference frequency (Figure 2B). This method works because noise at frequencies other than ν is rejected and does not affect the measurement [27]. Throughout this article, *relative amplitude* refers to *A*(*x,y*)/*A*_1_ where *A*_1_ is signal amplitude at the reference frequency in the stimulated cell. At each point (*x,y*), relative amplitude values remained stable for tens of seconds during carrier wave delivery to cell 1, but decreased rapidly with distance from this cell (Figure 2C and D). *At the single pixel level*, the standard deviation *σ* of the signal *A*(*x,y*) returned by the digital phase–sensitive detector scaled correctly as the square root of the number *N* of integration cycles (Figure 2E). Note that *σ* ≈ 2.2 mV at *N* = 1 and *σ* ≈ 0.5 mV at *N* = 25; reaching sub–mV sensitivity required *N* ≥ 5.

**Figure 3:**
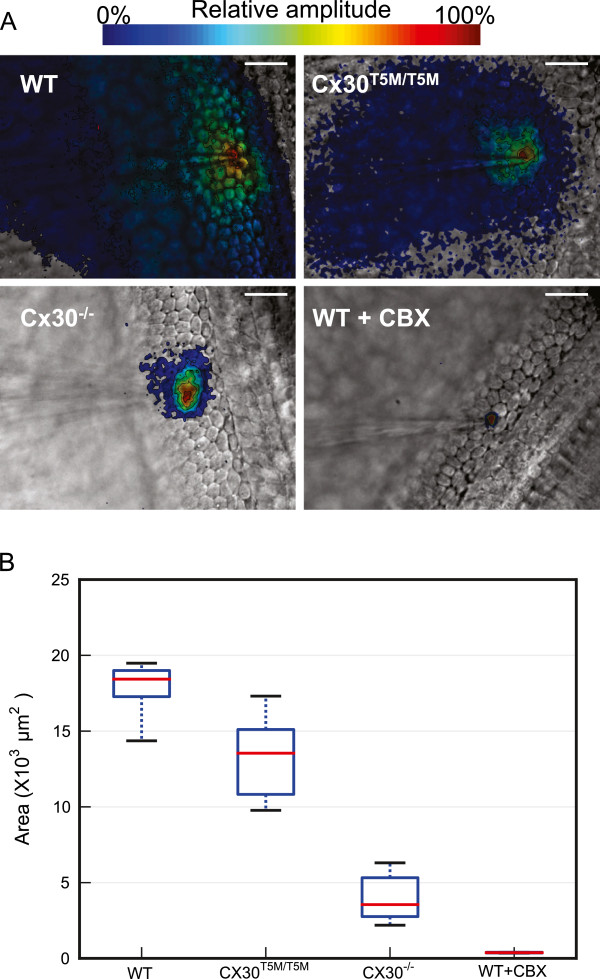
**Direct readout of network connectivity by large–scale optical recordings of Vf2.1.Cl fluorescence responses to a 0.5 Hz 35 mV carrier wave. (A)** Representative false–color images showing the spatial distribution of Vf2.1.Cl relative amplitude signals in cultures from P5 wild type (top left), connexin30^T5M/T5M^ (top right) and connexin30^−/−^ (bottom left) mice; the bottom right image refers to a wild type culture in which gap junction channels were blocked by 20 min incubation with CBX (100μM); in this image, the area with a residual relative amplitude signal (226 μm^2^) is very close to the average area of a single cell in this part of the culture (210±7 μm^2^, *n* = 10 cells); scale bars, 25 μm. **(B)** Suprathreshold area distributions shown in box plot form; see main text for details.

To estimate cell network extension, we computed *A*(*x,y*) by integrating Vf2.1.Cl signals over *N* = 5 carrier wave cycles (Figure 3). This approach permitted us to discriminate rapidly (10 s per recording) network connectivity of wild type cultures (Figure 3A, top left) from that of genetically modified connexin30^T5M/T5M^ (top right) and connexin30^**−/−**^ (bottom left) cultures [28]. Incubating wild type cultures for 20 minutes in 100 μM CBX confined the Vf2.1.Cl signal to the stimulated cell (bottom right), indicative of junctional conductance (*g*_*j*_) collapse over the entire network.

For statistical comparison, we increased the precision of these steady–state measurements by integrating Vf2.1.Cl signals over *N* = 25 carrier wave cycles (50 s per recording) and measured the culture area where *A*(*x,y*) exceeded an arbitrary threshold value corresponding to 2*σ* ≈ 1.0 mV (suprathreshold area; pooled results are summarized in Figure 3B). Compared to wild type cultures, suprathreshold areas in connexin30^T5M/T5M^ and connexin30^**−/−**^ cultures were significantly shifted towards lower values (*p* = 0.03 and *p* = 0.006, respectively; Mann–Whitney *U* test; *n* = 5 cultures for each genotype). In wild type cultures, the lower quartile, the median, and upper quartile of suprathreshold area values were respectively: 17230, 18430, 18970 μm^2^; the corresponding values in connexin30^T5M/T5M^ cultures were: 10730, 13550, 15100 μm^2^; finally, in connexin30^**−/−**^ cultures, they were: 2730, 3550, 5300 μm^2^.

### A simple resistive network model accounts for the spatial dependence of Vf2.1.Cl signals

**Figure 4:**
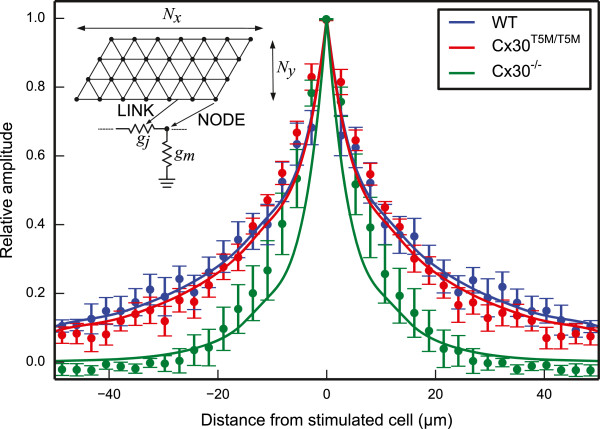
**Data fit by a simple resistive network model that reflects the anatomy.** The inset shows model scheme. Simulations were performed using the *ngspice* software (http://ngspice.sourceforge.net). *N*_*x*_ = 45 and *N*_*y*_ = 15 indicate the number of rows and columns in the grid, respectively. Each node represents one cell and each link represents a resistive connection between adjacent nodes. The patch pipette connected to cell 1 was simulated as a variable voltage source connected to one node of the grid through an access resistance *R*_*a*_ = 7.8 MΩ (not shown). A single value for membrane conductance (*g*_*m*_ = 8.3 nS) and junctional conductance (*g*_*j*_) were used throughout the network. *g*_*j*_ was left as the only free parameter in the simulations and its value was derived using a maximum–likelihood algorithm.

To gain further insight into the spatial dependence of the data shown in Figure 3, we modeled the cell network as a collection of nodes (individual non–sensory cells) forming an hexagonal mesh that reflects the anatomy [29]. In this model, nodes were coupled by resistive links with identical junctional conductance *g*_*j*_ . Each node was also connected to ground by a resistor with conductance *g*_*m*_ representing cell membrane (Figure 4). We pooled data from *n* = 5 cultures for each genotype at equal distances from the stimulated cell along the coiling axis of the cochlea and plotted the result versus this distance. Finally, we obtained least–square fits to these averaged data using the network model with *g*_*j*_ as the only free parameter. The results were: *g*_*j*_ = 206 nS for wild type, 177 nS for connexin30^T5M/T5M^ and 19 nS for connexin30^**−/−**^ cultures.

### Application to network dynamics

**Figure 5:**
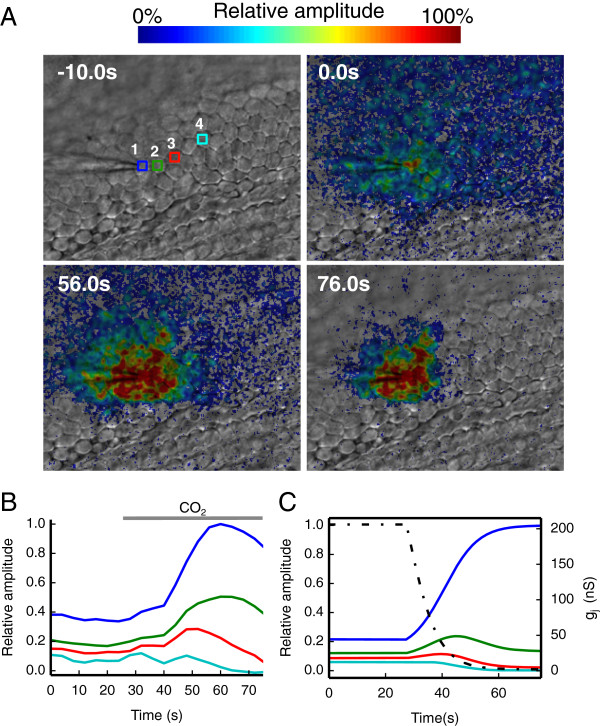
**Effect of cell uncoupling by CO**_**2**_**. (A)** Four selected frames from the same image sequence showing the progressive reduction of the number of cells coupled to the stimulated cell before and during exposure to 100% CO_2_; the top left image was captured 10.0 s before the delivery of the carrier wave stimulus to cell 1; CO_2_ delivery started at 25 s and was maintained thereafter; scale bar, 25 μm. **(B)** Time course of pixel averages from the color−coded ROIs shown in **(A)**. **(C)** Numerical simulation of the electrical uncoupling process; the effect was mimicked by rapidly decreasing the value of junctional conductance *g*_*j*_ (black dashed line) in the network model of Figure 4 from an initial value of 206 nS to 2 nS.

In patho–physiological conditions, gap junction networks are dynamically regulated by a variety of mechanisms that govern connexin channel permeability and gating [1,7,8,20,30]. Our next goal was to track dynamical changes in cell network connectivity by applying a digital phase–sensitive detector to Vf2.1.Cl signals. For this series of recordings, we limited time integration to *N* = 4 carrier wave cycles while transiently superfusing cochlear cultures with an extracellular medium saturated with 100% CO_2_ to produce carbonic acid (H_2_CO_3_). In its non−dissociated form H_2_CO_3_ is membrane permeable and causes a rapid and reversible closure of gap junction channels [7,30]. This manipulation led to a reduction in the number of cells coupled to the stimulated cell, accompanied by a transient increase in Vf2.1.Cl fluorescence in the neighborhood of this cell (Figure 5A, B and Additional file 2: Movie S2). To mimic the time course of the events shown in Figure 5A, B, we simply assumed that the *g*_*j*_ of the network model represented in Figure 4 undergoes a time−dependent exponential decrease from 206 nS to 2 nS with a time constant of 7 s (Figure 5C).

### Application to immortalized cell lines

**Figure 6:**
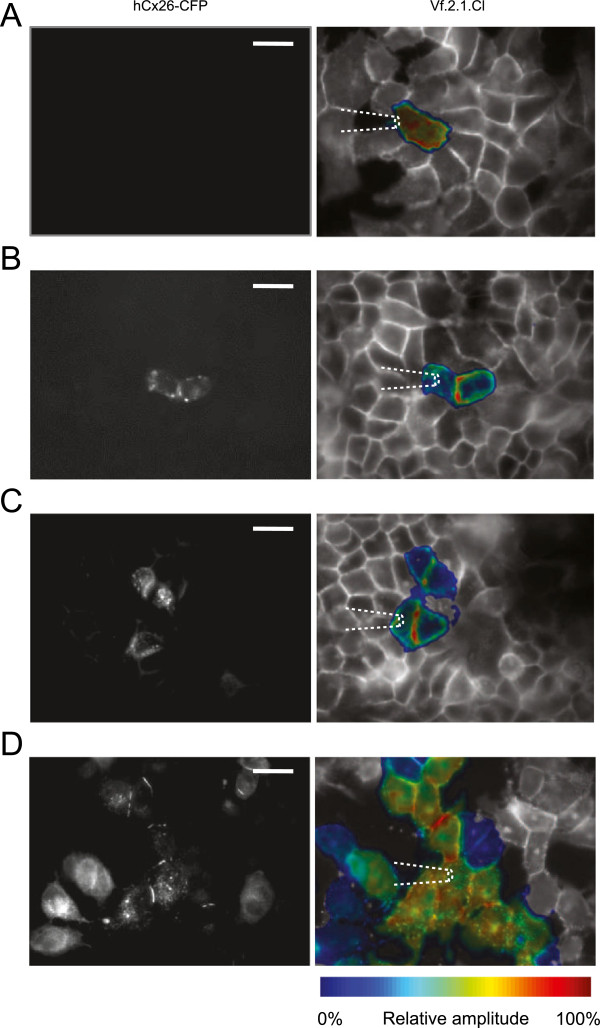
**Optical readout of network connectivity in HeLa cells loaded with Vf2.1.Cl. (A)** HeLa parental cells. **(B**, **C**, **D)** Transiently transfected HeLa cells showing increasing amounts of interconnectivity provided by hCx26–CFP gap junction channels; images of CFP fluorescence emission from chimerical proteins are shown at left, the corresponding Vf2.1.Cl relative amplitude data at right (integration cycles *N* = 25); scale bars, 25 μm.

A number of gap junction communication studies are performed in expression systems and/or immortalized cell lines. To demonstrate the applicability of the method highlighted above to this important area of research, we used a clone of HeLa cells virtually devoid of connexins (see Methods) that were either left untreated (HeLa parental) or transiently transfected with a construct expressing human connexin26 fused in tandem with a cyan fluorescent protein (CFP) reporter (hCx26–CFP) [20]. These chimerical proteins localized to the plasma membrane at regions of contact between adjacent cells and also formed distinct fluorescent puncta in the cytoplasm, as previously described [20,31,32]. Confluent HeLa cell cultures were loaded with the Vf2.1.Cl dye and subjected to the same patch–clamp protocol used in organotypic cochlear cultures. In HeLa parental cultures, the Vf2.1.Cl signal remained confined to the stimulated cell (Figure 6A). In transfected cultures, the Vf2.1.Cl signal displayed variable degrees of cell–to–cell spreading, reflecting the number of transfected cells connected to the stimulated cell by hCx26–CFP gap junction channels (Figure 6B, C and D).

## Discussion

We report here the application of the novel Vf2.1.Cl fluorescent sensor [18] to voltage imaging in cell networks coupled by gap junction channels. We focused our attention on non−sensory cell networks of the developing cochlea and used organotypic cultures from P5 mice as model system. Our *in situ* calibration yielded an estimated responsivity of 23 ± 3 % Δ*F/F*_0_ per 100 mV change of the cell membrane potential *V*_*m*_ (Figure 1), in substantial agreement with measurements performed in HEK293 cells (26% per 100 mV) [18]. The limited dispersion of the data in Figure 1C, which were acquired under different *F*_0_ conditions, indicates that the responsivity is fairly independent of the loading conditions. However, *F*_0_ does affect the signal−to−noise ratio, which is ultimately dictated by fluctuations in the number of collected photons (see, e.g. Ref. [26]), and consequently also the sensitivity of the measurement.

The Vf2.1.Cl signal tracks the membrane potential with no detectable delay [18], a highly desirable feature that distinguishes this dye from the substantially slower voltage sensors based on fluorescent proteins. The use of these proteins is also hindered by the necessity of transfecting/transducing target cells with a suitable expression vector [33]. In contrast, Vf2.1.Cl loads readily (15 min) and our use of a digital phase–sensitive detector (see Methods and Figure 2) allowed us to rapidly map cellular connectivity over vast network areas (Figures 3, 4, 5 and 6). With our methodology, the time required for data collection is a multiple of the carrier wave period (2 s in our conditions). Increasing the integration interval slows down the acquisition (i.e. it reduces the temporal resolution of dynamical measurements such as those presented in Figure 5) but reduces fluctuations (Figure 2E) and thus increases both sensitivity and precision (defined as the degree to which repeated measurements under unchanged conditions show the same result).

By integrating the Vf2.1.Cl signal over *N* = 5 carrier wave cycles (10 s), we detected intercellular connectivity down to (at least) 10^th^ order cells (i.e. to cells that were separated from the stimulated cells by a linear sequence of 10 adjacent neighbors), in wild type cultures. For comparison, microinjection experiments with fluorescent tracers that permeate cochlear gap junction channels (e.g. calcein, a relatively large permeant tracer that barely fits into the pore of connexin26 channels [34]) require typically 4 minutes to allow dye transfer to 3^rd^ or 4^th^ order cells (e.g., see Supplementary Figure 6 of ref. [22]; an example of a microinjection experiment performed in this preparation with the widely used fluorescent tracer Lucifer Yellow is shown in Additional file 3: Movie S3). Comparable time intervals are necessary to assay gap junction communication by fluorescence recovery after photobleaching (gap–FRAP) [35] (see, e.g. Figure 5 of ref. [36]).

The exact stoichiometry of cochlear gap junction channels in terms of connexin26 and connexin30 subunits is not known. Single channel currents from HeLa cells overexpressing either connexin26 or connexin30 homomeric channels yielded respectively values of 115 pS and 160 pS for the unitary conductance γ [20,32]. A study in HeLa cells co–transfected with the cDNA of both proteins, and thus presumably forming heteromeric/heterotypic channels, reported γ values in a comparable range of 110–150 pS [37]. The junctional conductance *g*_*j*_ = 206 nS we obtained by fitting wild type culture data in Figure 3 with the resistive network model of Figure 4 suggests that cochlear non−sensory cells are already well coupled at P5, by as many as *N*_open_ = *g*_*j*_ /γ = 1300 to 1800 open channels per cell pair. An older study in isolated pairs of supporting cells of the adult guinea pig organ of Corti reported that junctional conductance may exceed non−junctional conductance by three orders of magnitude and, at least in some cell pairs, *g*_*j*_ was as large as 1 μS [38] corresponding to *N*_open_ ~ 10^4^. We are not aware of structural investigations performed in the developing cochlea. However, Forge et al. [39] noted that gap junction plaques in the supporting cells of the mature cochlea are “enormous” and they often occupy a major fraction of the plasma membrane between two adjacent cells (from 25% to almost 100% in pillar cells). From their freeze fracture studies, Forge et al. concluded that plaques containing about 10^4^ channels are not rare and some may even contain 10^5^ channels, such as those coupling inner pillar cells in the longitudinal direction. Thus our *g*_*j*_ estimate is not in contrast with the proposal that only about 10% of channels within a plaque are in the open state [40-42].

Data in Figure 3 show a 27% and 80% reduction in the median suprathreshold area respectively for connexin30^T5M/T5M^ and connexin30^−/−^ cultures relative to wild type cultures. Our resistive network model suggests that these reduced areas correspond to a *g*_*j*_ decrease of 14% and 91% for connexin30^T5M/T5M^ and connexin30^−/−^ cultures, respectively. We previously reported massive down–regulation of connexin26 in the developing organ of Corti of connexin30^−/−^ mice [36]. Connexin26 is similarly reduced, to 10% of the wild type level, in the cochlea of adult connexin30^−/−^ mice. These findings complement and extend our prior work [28], which highlighted a significant reduction in the level of dye coupling in connexin30^T5M/T5M^ cultures, whereas dye coupling was absent in connexin30^−/−^ cultures. We also showed that adult connexin30^T5M/T5M^ mice, when probed by auditory brainstem recordings, exhibit a mild but significant increase in their hearing thresholds, of about 15 dB at all frequencies [28]. By contrast, connexin30^−/−^ mice are profoundly deaf [28,43]. The present experiments and our previous work confirm cochlear organotypic cultures as an attractive test ground to explore the intricacies of connexin expression regulation and function. In addition, our findings support the notion that connexin30^−/−^ mice are a model for humans in which large deletions in the DFNB1 locus lead to down–regulation of both *GJB6* and *GJB2* and to profound deafness [13].

It is well known that electrical conductance and permeability to solutes other than small inorganic ions are not directly related [8,20,44]. Even the junctional permeability to fluorescent probes may not be directly related to electrical coupling [28,45]. We believe that the methodology described in the present article may help clarifying this complex relationship when used in combination with other complementary techniques, particularly those that (i) provide a direct estimate of the unitary permeability to signaling molecules [7] and (ii) aid data interpretation by the use of Molecular Dynamics [34].

## Conclusions

Here we present a combined electrophysiological and optical approach to visualize rapidly and quantify connectivity in cell networks coupled by gap junctions. Our digital phase–sensitive detector of Vf2.1.Cl fluorescence emission allows greater sensitivity and better time resolution compared to classical tracer–based techniques, and permitted us to track dynamically intercellular connectivity down to the 10^th^ order in non−sensory cell networks of the developing cochlea. Despite the fact that the results shown here were obtained in specific cell models (cochlear non−sensory cells, HeLa cells) we believe that our method is of general interest and can be seamlessly extended to a variety of biological systems, as well as to other connexin−related disease conditions [10-12].

## Methods

### Reagents and drugs

Vf2.1.Cl [18] was provided by Roger Y. Tsien (University of California, San Diego). Carbenoxolone (CBX), pluronic F–127, Hanks’ balanced salt solutions (HBSS) and the salts used to prepare solutions were purchased from Sigma–Aldrich. Lipofectamine, Dulbecco’s modified Eagle’s medium (DMEM/F12) and fetal bovine serum (FBS) were purchased from Life Technologies. Cell Tak was purchased from Becton Dickinson.

### Cochlear organotypic cultures

Cochleae were dissected from P5 mouse pups in ice−cold Hepes buffered (10 mM, pH 7.2) HBSS, placed onto glass coverslips coated with 185 μg/ml of Cell Tak and incubated overnight at 37°C in DMEM/F12 supplemented with FBS 5%.

### HeLa cells

A clone of HeLa cells essentially devoid of connexins was provided by Klaus Willecke (University of Bonn, Germany) and cultured according to standard procedures. Twenty four hours after plating, a lipofectamine transfection system was used to transiently transfect these communication–incompetent HeLa cells with hCx26–CFP, a previously described human connexin26 construct tagged with the cyan fluorescent protein (CFP) at its carboxyl terminal end [20].

### Electrophysiology and fluorescence imaging

All experiments were performed at room temperature (22–25°C). Cochlear or HeLa cell cultures were transferred to the stage of an upright wide–field fluorescence microscope (BX51, Olympus) and continually superfused with EXM, an extracellular medium containing (in mM): NaCl 138, KCl 5, CaCl_2_ 2, NaH_2_PO_4_ 0.3, KH_2_PO_4_ 0.4, Hepes−NaOH 10, d−glucose 6 (pH 7.2, 300 mOsm). Glass capillaries for patch clamp recordings were formed on a vertical puller (PP–83, Narishige, Japan) from 1.5−mm outer diameter borosilicate glass (G85150T–4, Warner Instruments) and filled with an intracellular solution containing (in mM): KCl 134, NaCl 4, MgCl_2_ 1, HEPES 20, EGTA 10 (adjusted to pH 7.3 with KOH, 290 mOsm) and filtered through 0.22 μm pores (Millipore). Pipette resistances were 3–4 MOhm when immersed in the EXM bath. For whole−cell (paired) patch clamp recordings, cell 1 was maintained under voltage clamp conditions with a patch clamp amplifier (Model 2400, AM Systems) while cell 2 was kept under current clamp conditions with a second amplifier (EPC−7, HeKa). Current and voltage were filtered at 3 kHz by an 8 pole Bessel filter and sampled at 20 kHz using a standard laboratory interface (Digidata 1440A, Molecular Devices) controlled by the PClamp 10 software (Molecular Devices).

To visualize hCx26–CFP, transfected HeLa cells were illuminated by light from a 385 nm LED (M385L2, Thorlabs) passing through a D390/70X filter (Chroma) and directed onto the sample through a 440 dclp dichromatic mirror (Chroma) while CFP emission was selected by an ET480/40M filter (Chroma).

For voltage imaging, cochlear or HeLa cell cultures were incubated for 15 min at 37°C in EXM supplemented with Vf2.1.Cl (200 nM) and pluronic F–127 (0.1% w/v), thereafter cultures were continually superfused with EXM. Vf2.1.Cl fluorescence was excited by light from a 470 nm LED (M470L2, Thorlabs) passing through a BP460–480 filter (Olympus) and directed onto the sample through a 515 dcxr dichromatic mirror (Chroma) while Vf2.1.Cl fluorescence emission was selected by an ET535/30M filter (Chroma). All fluorescence images were formed by a 60× water immersion objective (NA 1.0, Fluor, Nikon) and projected on a scientific–grade CCD camera (SensiCam; PCO AG) controlled by software developed in the laboratory. Image sequences of Vf2.1.Cl fluorescence were acquired continuously at 10 frames per second with 100 ms exposure time. To synchronize image acquisition and electrical recordings, we sampled the 5 V pulse (FVAL) that signals active exposure of the CCD camera [46]. Vf2.1.Cl signals were measured as relative changes of fluorescence emission intensity (Δ*F/F*_0_), where *F*_0_ is prestimulus fluorescence, *F* is fluorescence at time *t* and Δ*F* = *F* – *F*_0_.

Miller et al. reported that Vf2.1.Cl and other PeT–based voltage indicators have a slower rate of bleaching and are less toxic than the FRET–based dyes [18]. We did not make a direct comparison between these two classes of indicators. However, in our hands patch clamp recordings from cochlear non–sensory cells in Vf2.1.Cl loaded cultures were stable for tens of minutes during continuous illumination with the LED used to excite dye’s fluorescence. In addition, we did not notice any visible sign of cellular degeneration.

### Image processing

Vf2.1.Cl fluorescence image sequences were stored on disk and processed off–line using the Matlab R2011a software package (The MathWorks, Inc.) as described hereafter. Following electrical stimulation of cell 1 with a carrier wave at frequency *ν*, each image was preprocessed by applying a 3–by–3 mean spatial filter to reduce acquisition noise. To correct for photobleaching, we first estimated its time course by performing a low order polynomial fit to the (Δ*F/F*_0_)(*t*; *x*, *y*) data at each pixel location (*x,y*); the fitting function *P*(*t*; *x*,*y*) was then subtracted from the (Δ*F/F*_0_)(*t*; *x*,*y*) signal, yielding an effectively high–pass filtered trace

$$f\left(t;x,y\right)=\frac{\mathrm{\Delta F}\left(t;x,y\right)}{F_{0}\left(x,y\right)}-P\left(t;x,y\right)$$

The purpose of using a phase–sensitive detector is to extract the signal amplitude *A*(*x,y*) from the preprocessed single pixel signal

$$f\left(t;x,y\right)=A\left(x,y\right)\cos\left(2\mathrm{\pi \nu t}-\theta \right)$$

where *θ* is a constant phase delay [27]. We performed the extraction procedure in two steps:

Step 1: demodulation. *f*(*t; x,y*) was multiplied by two orthogonal reference signals

$$V_{1}^{\mathrm{ref}}\left(t\right)=\cos\left(2\mathrm{\pi \nu t}\right)$$

$$V_{2}^{\mathrm{ref}}\left(t\right)=\sin\left(2\mathrm{\pi \nu t}\right)$$

(see Figure 2B) yielding two output signals of the form

$$\begin{array}{c} f_{1}\left(t;x,y\right)=V_{1}^{\mathrm{ref}}(t)\cdot f(t;x,y)=A(x,y)\cos(2\mathrm{\pi \nu t}-\theta )\cos(2\mathrm{\pi \nu t})\\ f_{2}\left(t;x,y\right)=V_{2}^{\mathrm{ref}}(t)\cdot f(t;x,y)=A(x,y)\cos(2\mathrm{\pi \nu t}-\theta )\sin(2\mathrm{\pi \nu t}) \end{array}$$

Considering the trigonometric identities

$$\begin{array}{l} \cos\left(a\right)\cos(b)=\frac{1}{2}[\cos(a-b)+\cos(a+b)]\\ \cos\left(a\right)\sin(b)=\frac{1}{2}[\sin(a+b)-\sin(a-b)] \end{array}$$

the two output signals can be written as

$$\begin{array}{l} f_{1}\left(t;x,y\right)=\frac{1}{2}A(x,y)[\cos(\theta )+\cos(2\pi (2\nu )t-\theta )]\\ f_{2}\left(t;x,y\right)=\frac{1}{2}A(x,y)[\sin(2\pi (2\nu )t-\theta )+\sin(\theta )] \end{array}$$

and are seen to consist of a DC signal proportional to the amplitude *A*(*x,y*) of the original function *f*(*t; x,y*) and a time–dependent component with frequency 2*ν*.

Step 2: Amplitude estimation. The time–dependent component was filtered out by time integration of *f*_1_(*t; x,y*) and *f*_2_(*t; x,y*). In the absence of noise, integration over a single carrier wave cycle would yield the DC components of *f*_1_(*t; x,y*) and *f*_2_(*t; x,y*):

$$\begin{array}{c} a_{1}\left(x,y\right)=\frac{1}{2}\;A(x,y)\;\cos(\theta )\\ a_{2}\left(x,y\right)=\frac{1}{2}\;A(x,y)\;\sin(\theta ) \end{array}$$

In practice, integration is better performed over a number *N* of cycles to reduce contributions from various noise sources (see Figure 2E). Finally, the amplitude of *f*(*t; x,y*) was computed as

$$A\left(x,y\right)=2\sqrt{{\left[a_{1}\left(x,y\right)\right]}^{2}+{\left[a_{2}\left(x,y\right)\right]}^{2}}$$

The reference level *A*(*x,y*) = 0 was set by applying the above algorithm to the pre–stimulus trace (i.e. to the trace segment that preceded cell 1 stimulation by the carrier wave).

### Statistical analysis

Means are quoted ± standard error of the mean (s.e.m.) and p–values are indicated by letter *p*. Statistical comparisons were made using the Mann–Whitney *U* test [47] and *p* < 0.05 was selected as the criterion for statistical significance.

### Animal handling

Animal handling was approved by the Ethical Committee of Padua University (Comitato Etico di Ateneo per la Sperimentazione Animale, C.E.A.S.A.) project n. 54/2009, protocol n. 51731.

## Abbreviations

VF: VoltageFluor; GJB2: Gene encoding gap junction beta–2 protein; GJB6: Gene encoding gap junction beta–6 protein; CBX: Carbenoxolone; CFP: Cyan fluorescent protein; FRAP: Fluorescence recovery after photobleaching; ROI(s): Region(s) of interest.

## Competing interests

The authors declare that they have no competing interests.

## Authors’ contributions

FM and FC respectively designed and performed the experiments; FC analyzed data; FM wrote the paper. Both authors read and approved the final manuscript.

## Supplementary Material

Additional file 1: Movie S1 Effect of carrier wave stimulation. This video shows raw fluorescence signals from a P5 wild type cochlear organotypic culture loaded with Vf2.1.Cl; a patch pipette entering from the left delivers a sinusoidal voltage stimulation at 0.5 Hz (carrier wave) to a cell maintained under whole–cell conditions. Click here for file

Additional file 2: Movie S2 Effect of CO_2_ application. This is the video sequence from which frames in Figure 5 were extracted. It shows processed fluorescence signals from a cochlear organotypic culture loaded with Vf2.1.Cl during application of CO_2_ (see main text for details). Click here for file

Additional file 3: Movie S3 Lucifer Yellow delivery via patch pipette to a non–sensory cell of the lesser epithelial ridge. This video sequence was captured from a P5 wild type cochlear organotypic culture while delivering Lucifer Yellow dissolved at a concentration of 225 μM in the intracellular solution described in the Methods. Note that the patch pipette used for dye microinjection had the same physical characteristics (mouth diameter, electrical resistance) of those utilized for the delivery of the carrier wave signals of our digital phase–sensitive detector. Click here for file
